# Adult-onset chronic recurrent multifocal osteomyelitis: A case report of a rare entity

**DOI:** 10.12669/pjms.40.8.8765

**Published:** 2024-09

**Authors:** Huzefa Jibril, Mehmood Riaz, Syed Ahsan Ali

**Affiliations:** 1 Dr. Huzefa Jibril, MBBS. Department of Medicine, The Aga Khan University Hospital, Karachi, Pakistan; 2 Dr. Mehmood Riaz, FCPS. Department of Medicine, The Aga Khan University Hospital, Karachi, Pakistan; 3 Dr. Syed Ahsan Ali, FCPS. Department of Medicine, The Aga Khan University Hospital, Karachi, Pakistan

**Keywords:** Back pain, Chronic recurrent multifocal osteomyelitis, Low back pain, Janus Kinase Inhibitors

## Abstract

**Background:**

Chronic recurrent multifocal osteomyelitis is a rare autoimmune disorder causing inflammatory joint lesions. It has an estimated prevalence of 1-2 per million while adult-onset disease constitutes only 6.3% of patients.

**Case report:**

We present a case of a 44 years old male who presented to the rheumatology clinic with lower back pain for twelve years. Magnetic resonance imaging of the lumbosacral spine showed ovoid areas of abnormal signal intensities along superior and inferior endplates of multiple vertebrae of the dorsolumbar and sacral spine. Computed tomography guided biopsy of L4 vertebrae was done. Histopathology revealed linear cores of degenerating fibrocartilage focally exhibiting small spicules of mineralized bone and fibro-collagenous tissue. He initially did not respond to traditional therapy. His symptoms improved with the addition of a Janus Kinase inhibitor. To the best of our knowledge, this is the first case of chronic recurrent multifocal osteomyelitis to be reported from Pakistan.

## INTRODUCTION

Chronic recurrent multifocal osteomyelitis (CRMO), also called Chronic Non-bacterial Osteomyelitis (CNO), is a rare autoimmune disease characterized by inflammatory joint lesions.^1^ It is estimated to have a prevalence of approximately 1-2 per million^2^ and is exceptionally rare in adults, occurring only 6.3% of the time.^3^

The median age of diagnosis in adults is 32.5 [22-56] years while a family history of chronic immune mediated disease is present in 20%.^4^ Complications include bone fractures and pain.^5^ Along with musculoskeletal problems, muco-cutaneous, gastrointestinal, lymphoid, ocular, and cardiac manifestations have also been reported.^3^

We present a case of an adult patient with CRMO who presented with lower back pain for the last twelve years. He initially did not respond to traditional therapy. His symptoms then improved with a Janus Kinase (JAK) inhibitor. To our knowledge, this is the first case of CRMO to be reported from Pakistan.

## CASE REPORT

A 44-year-old male presented to the rheumatology clinic with lower back pain for the last twelve years. The pain was severe in nature, radiating upwards, and worsening with exertion. In the last four to five months, his pain had worsened. He had no complaints of pain in either the small or large joints of the upper or lower limbs. He did not have upper or lower limb weakness and urinary or fecal incontinence. He denied any family history of rheumatological disease. Neurological examination was unremarkable. There was no joint swelling, tenderness or restriction of movement in any joint. The rest of the systemic examination was also unremarkable. He did not have any cutaneous manifestations.

**Fig.1:**
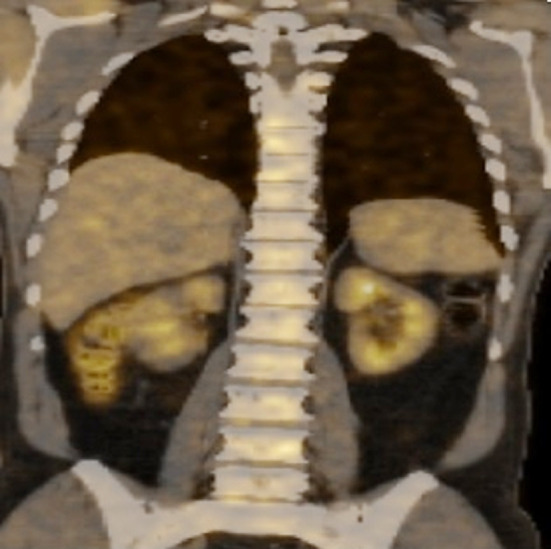
PET-CT showing low FDG avid small areas of osteolytic and patchy sclerosis involving multiple dorso lumbar vertebrae. PET: Positron emission tomography – computed tomography, FDG: Fluorodeoxyglucose

**Fig.2:**
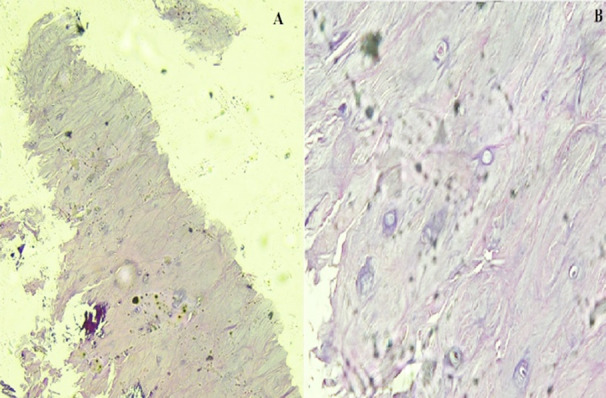
Histopathology appearance of specimen from L4 vertebral showing areas of degenerating fibrocartilage focally exhibiting small spicules of mineralized bone and fibrocollagenous tissue at 10X (A) and 40X (B).

Complete blood count demonstrated a hemoglobin of 11.1 g/dl, hematocrit of 35.0%, total leukocyte counts of 7.2 × 10^9^/L and platelets of 572 × 10^9^/L. His ESR was 116 mm/1st hour while creatinine was 0.9 mg/dl. C-reactive protein was 39.70 mg/L. Serum protein electrophoresis was normal and the albumin/globulin ratio was 1.1. A bone scan showed focal areas of increased tracer uptake involving the right 6^th^-7^th^ ribs anteriorly and right 7^th^-8^th^ ribs posteriorly and L2 vertebrae. Magnetic resonance imaging (MRI) of the lumbosacral spine with and without contrast was done showing ovoid areas of abnormal signal intensities (hypo-intense on T1, hyper-intense on T2, peripheral enhancement on post contrast T1-weighted images) and along superior and inferior endplates of multiple vertebrae of the dorso-lumbar and sacral spine. Positron emission tomography - computed tomography (PET-CT) demonstrated low fluorodeoxyglucose (FDG) avid small areas of osteolytic and patchy sclerosis involving left side of body of sternum, C2 and multiple dorso-lumbar vertebrae (Fig.1). Computed tomography (CT) guided biopsy of the L4 vertebrae was done and histopathology revealed linear cores of degenerating fibrocartilage focally exhibiting small spicules of mineralized bone and fibro-collagenous tissue (Fig.2).

The clinical score proposed by Jansson et al. for our patient was 53 with a score ≥ 39 indicating a probable diagnosis with a nominal positive predictive value of 97%.^6^ An impression of vertebral CRMO was made. The patient was started on oral prednisolone 20 mg per day, oral methotrexate 10 mg per week and intravenous bisphosphonate. On follow up after a month his symptoms did not improve. Methotrexate was increased to 12.5mg per week and prednisolone gradually tapered to 5mg per day. He presented again a month later with worsening back pain and aches all over the body. On this visit he was given ketorolac intramuscularly and prednisolone was increased to 20mg per day. On follow-up after two weeks, his pain did not improve. After screening for tuberculosis and chronic viral hepatitis, he was started on oral tofacitinib 10mg per day and prednisolone was tapered gradually to 5mg per day while methotrexate was decreased to 10mg per week.

Five months later on follow-up, his symptoms were controlled with prednisolone (5mg per day), methotrexate (10mg per week) and tofacitinib (10mg per day). Prednisolone was discontinued while methotrexate and tofacitinib were continued. After eight months of follow up, his symptoms have remained controlled. On last follow up, his ESR was 23 mm/1^st^ hour and C-reactive protein was 4.98 mg/L.

Written informed consent from the patient was taken for the report of this case and approval from the ethical review committee (ERC) of the university hospital was taken (ERC reference number 2023-9029-26320). Date September 02, 2023.

## DISCUSSION

Though the exact pathogenic mechanisms of CRMO are unknown, an imbalanced expression of pro-inflammatory cytokines such as interleukin-6 has been proposed.^5^ Adult onset CRMO is more common in females, while the onset of symptoms is between 33 to 37 years of age with bone pain the most common presenting symptom.^7^ Sternal and vertebral involvement is more common in adults, while clavicle and long bone metaphysis are more commonly involved in children.^7^ The imaging modality of choice is MRI, particularly with short tau inversion recovery (STIR) sequences, because multifocal bone lesions are more common than unifocal lesions.^7^ CRMO is a diagnosis of exclusion with hematological and bone malignancies and infectious osteomyelitis amongst the differentials.^8^

Non-steroidal anti-inflammatory (NSAID) drugs, systemic glucocorticoids, disease modifying anti-rheumatic drugs (DMARDs), and bisphosphonates are commonly used as initial treatment options.^9^ In contrast to the rates of remission observed in children (66.25%), the rates of remission achieved in adults are low (30%).^4^

Tofacitinib is a reversible inhibitor of JAK1, JAK2, JAK3 and to a lesser extent Tyrosine Kinase-2.^10^ In a recent review, tofacitinib has been shown to be a promising treatment option for patients with CRMO not responding to traditional therapy.^11^ The underlying mechanism may be by blocking JAK pathways which are used by cytokines to transmit signals such as interleukin-6.^12^ Other biologics that have been shown to be of benefit in patients with osteoarticular symptoms include tumor necrosis factor, interleukin-1, and interleukin-17 inhibitors.^11^

## CONCLUSION

Though a rare disease, CRMO should be considered in the differential diagnosis of lower back pain after common causes have been ruled out. Traditionally remission has been difficult to achieve. However, biologics such as JAK inhibitors are showing promising results.

### Author`s Contribution:

**HJ:** Manuscript writing.

**MR:** Conceptualization, follow-up of patient, review and final approval of manuscript.

**SAA:** Editing of manuscript.
